# Exosome inspired photo-triggered gelation hydrogel composite on modulating immune pathogenesis for treating rheumatoid arthritis

**DOI:** 10.1186/s12951-023-01865-8

**Published:** 2023-03-28

**Authors:** Ke Rui, Xiaoxuan Tang, Ziwei Shen, Chao Jiang, Qiugang Zhu, Shiyi Liu, Nan Che, Jie Tian, Jue Ling, Yumin Yang

**Affiliations:** 1 Institute of Medical Immunology, Affiliated Hospital of Jiangsu University, Zhenjiang, China; 2 Key Laboratory of Neuroregeneration, Co-innovation Center of Neuroregeneration, Jiangsu Clinical Medicine Center of Tissue Engineering and Nerve Injury Repair, Ministry of Education and Jiangsu Province, Nantong University, Nantong, China; 3 Department of Immunology, Jiangsu Key Laboratory of Laboratory Medicine, School of Medicine, Jiangsu University, Zhenjiang, China; 4 Department of Rheumatology, The First Affiliated Hospital of Nanjing Medical University, Jiangsu, China

**Keywords:** Rheumatoid arthritis, Autoimmune pathogenesis, T follicular helper cells, Exosomes, In-situ hydrogel

## Abstract

Although exosome therapy has been recognized as a promising strategy in the treatment of rheumatoid arthritis (RA), sustained modulation on RA specific pathogenesis and desirable protective effects for attenuating joint destruction still remain challenges. Here, silk fibroin hydrogel encapsulated with olfactory ecto-mesenchymal stem cell-derived exosomes (Exos@SFMA) was photo-crosslinked in situ to yield long-lasting therapeutic effect on modulating the immune microenvironment in RA. This in situ hydrogel system exhibited flexible mechanical properties and excellent biocompatibility for protecting tissue surfaces in joint. Moreover, the promising PD-L1 expression was identified on the exosomes, which potently suppressed Tfh cell polarization via inhibiting the PI3K/AKT pathway. Importantly, Exos@SFMA effectively relieved synovial inflammation and joint destruction by significantly reducing T follicular helper (Tfh) cell response and further suppressing the differentiation of germinal center (GC) B cells into plasma cells. Taken together, this exosome enhanced silk fibroin hydrogel provides an effective strategy for the treatment of RA and other autoimmune diseases.

**Supplementary Information:**

The online version contains supplementary material available at 10.1186/s12951-023-01865-8.

## Introduction

As a chronic immune-mediated disease, rheumatoid arthritis (RA) is characterized by synovitis and destruction of articular cartilage [1, 2]. Patients with RA are at higher risk for osteoporosis, cardiovascular disease and cancer, which results in significant social-economic problems [3]. Current therapeutic approaches, such as cytokine suppressive drugs and surgery, can only achieve symptom relief and slow down the process of joint destruction [4, 5]. Thus, development of a treatment modality against specific pathological environments for alleviating immune dysregulations and effectively protecting joint is necessary.

T follicular helper cells (Tfh), a newly discovered subset of CD4 ^+^ T cells, play a significant pathogenic role in RA [6–8]. Tfh can interact with germinal center B cells (GC B) to maintain their survival and promote their differentiation into plasma cells (PC) for antibody production upon antigenic challenge [9]. Mesenchymal stem cells (MSCs) can effectively modulate the immune response by secreting anti-inflammatory cytokines. However, suboptimal differentiation of MSCs in inflammatory environment and their fast clearance by the immune system greatly compromise the therapeutic efficacy of MSC therapy in RA [10]. Alternatively, exosomes, as an essential component of extracellular vesicles, can carry biological molecules such as proteins, lipids and RNAs, which are involved in intercellular communication pathways for regulating the immune response in vivo [11, 12]. Recently, we have demonstrated that olfactory ecto-mesenchymal stem cell-derived exosomes (OE-MSC-Exos) possess therapeutic effect on suppressing autoimmune pathogenesis, such as experimental Sjögren’s syndrome (ESS) and inflammatory bowel disease (IBD) [13, 14]. Although exosomes have exhibited potent immune modulatory functions, the short residence time in joint tissue limits their therapeutic efficacy and their therapeutic mechanism still remains unclear [15, 16].

In situ gelation system can be delivered to fill irregularities of target sites upon local microenvironment or external stimuli to reduce the friction between tissue surfaces in joints [17–19]. Combined with exosome therapy, in situ hydrogels also allow extended exosome release and protects them from enzymatic degradation [20–22]. Specially, photo-triggered gelation system that cross-linked under source of UV and visible light irradiation offers great opportunities to delivery exosomes in the site of interest, due to its mild gelation conditions with high spatial and temporal precision of the gelation process [23–26]. However, the poor biocompatibility and insufficient bioactivity of most synthetic polymers limits the efficacy of in situ hydrogel based therapeutic strategies. As a natural protein, silk fibroin based scaffolds with low immunogenicity exhibit excellent biocompatibility and favorable chondrocyte response for biomedical applications in cartilage tissue engineering [27–29]. Moreover, we previously demonstrated that the in situ silk fibroin hydrogel could successfully recruit immune cells to enhance the therapeutic effect on remodeling the immune microenvironment [30].

**Scheme 1:**
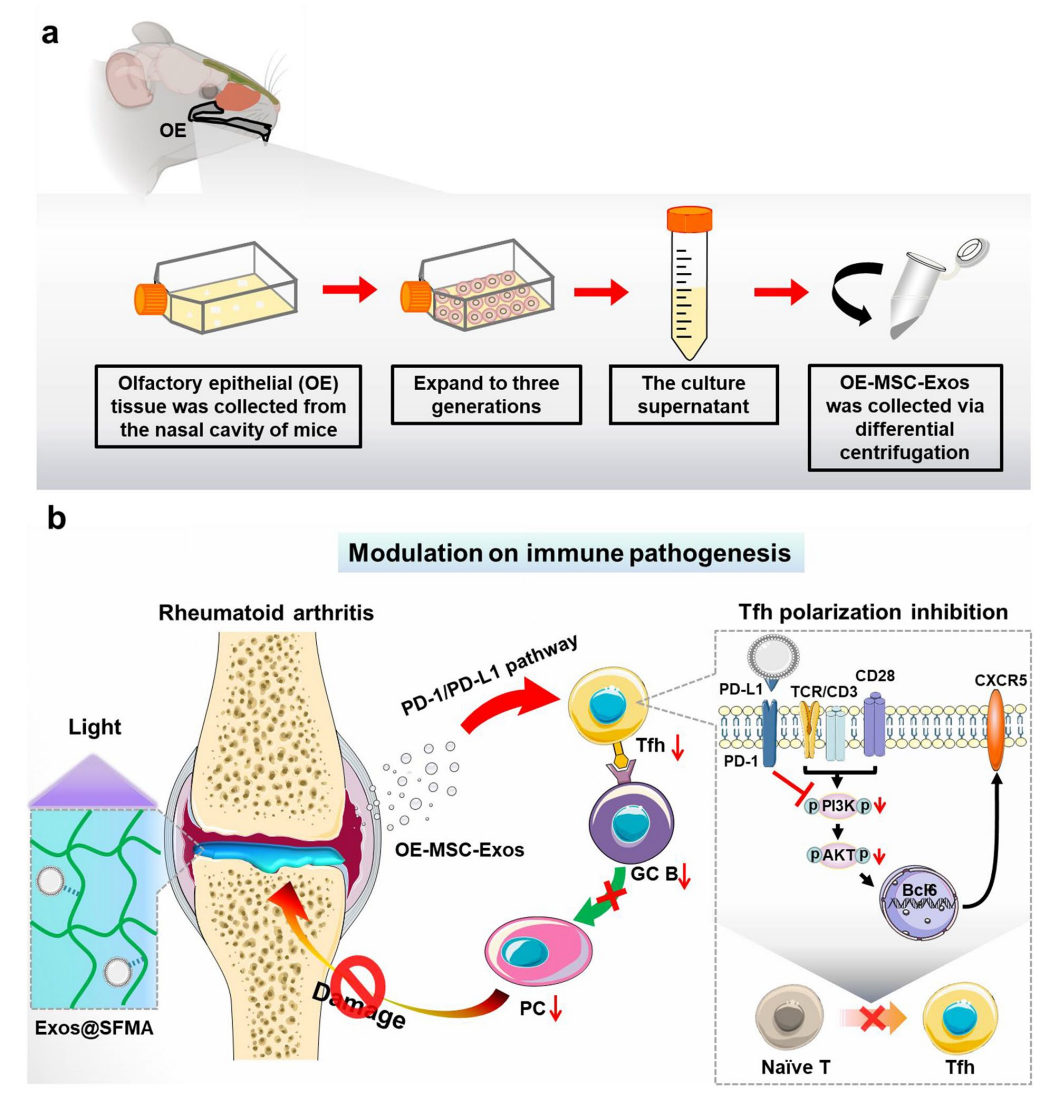
Schematic illustration of (a) isolation of exosomes from OE-MSCs obtained from olfactory lamina propria and (b) in situ gelation system improved exosome therapy for successfully modulating immune pathogenesis by inhibiting Tfh cell polarization and B cell development in RA.

Olfactory ecto-mesenchymal stem cells (OE-MSCs) can be isolated from the olfactory lamina propria and retain the potential for multidirectional differentiation [31, 32]. As the olfactory mucosa nerve tissue is permanently renewable and the nasal sheath is an open organ, OE-MSCs are easily accessible in every individual and have normal functions and renewability even in old persons, facilitating the OE-MSC-Exos based therapies [33]. In this study, an effective strategy on modulating immune pathogenesis against RA was developed by encapsulating OE-MSC-Exos into photo-cross-linkable silk fibroin hydrogel for in situ treatment of RA and protecting joints. The exosomes released from Exos@SFMA successfully inhibited Tfh cell polarization by expressing PD-L1 to down-regulate the PI3K/AKT pathway in T cells. Significantly, Exos@SFMA hydrogel holds excellent in vivo therapeutic effects on suppressing Tfh cell response and hindering development of GC B cells and plasma cells for treating RA (Scheme 1).

## Results and discussion

### Characterization of OE-MSC-Exos and their suppressive effects on tfh cell response in vitro

**Fig. 1:**
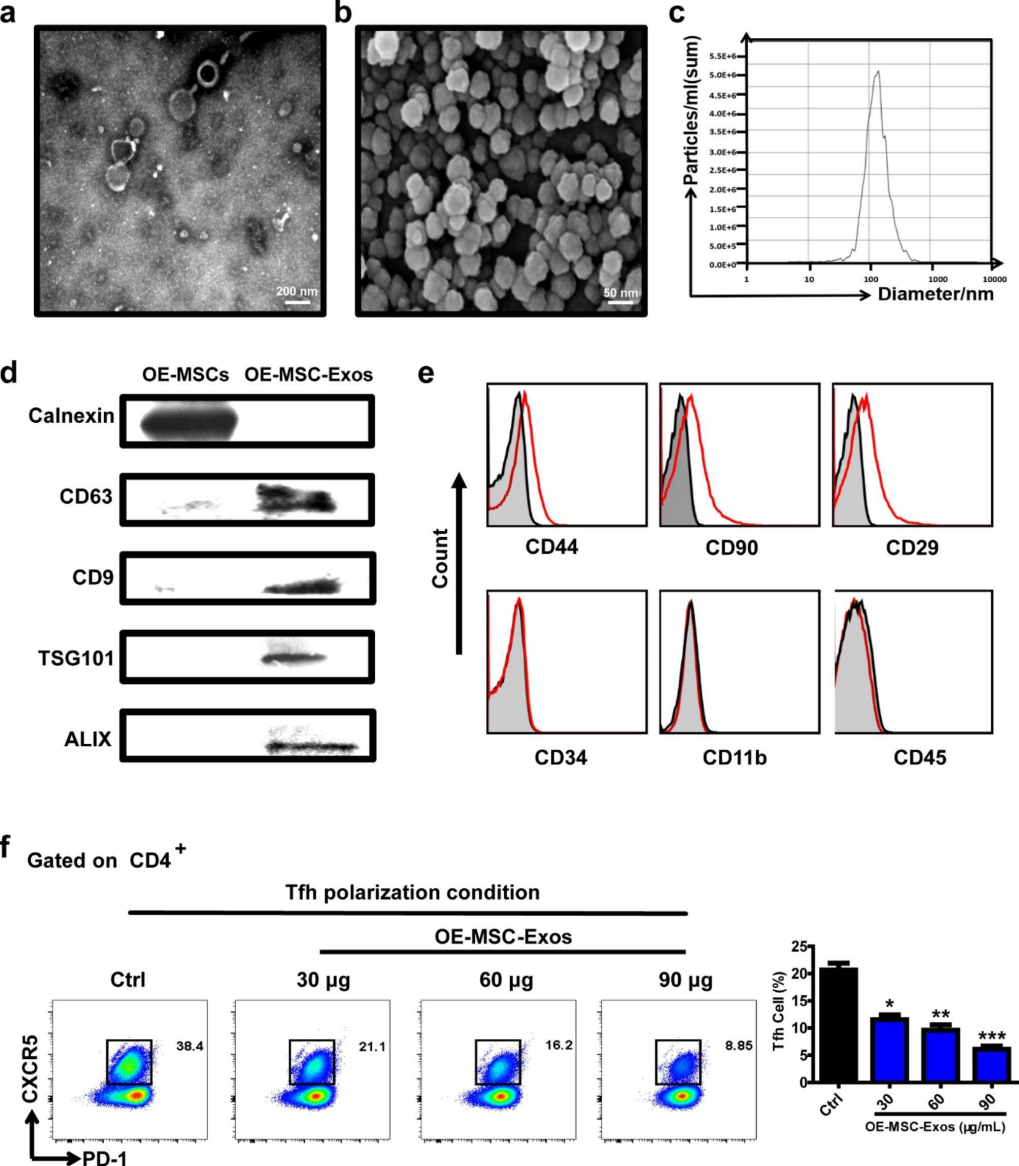
Isolation and characterization of OE-MSC-Exos and their suppression on Tfh cell responses in vitro. (a, b) Representative TEM (a) and SEM (b) images of exosomes derived from OE-MSCs. (c) The particle sizes of OE-MSC-Exos were determined by NTA. (d) Western blot analysis of CD63, CD9, TSG101, ALIX and Calnexin in OE-MSC-Exos. (e) The expression of typical identification markers of MSCs on the surface of OE-MSC-Exos. (f) Naïve CD4 + T cells from the spleen of C57BL/6 mice were cultured for 72 h in the presence of OE-MSC-Exos (30, 60, 90 µg/ml) under Tfh differentiation conditions and analyzed by flow cytometry assay. Values represent means ± S.D. (n = 3). *P < 0.05, **P < 0.01, ***P < 0.001

Initially, exosomes were isolated from the stem cell conditioned medium of olfactory ecto-mesenchymal stem cells (OE-MSCs) using the ultracentrifugation method [13]. The morphology and diameter of exosomes were investigated using transmission electron microscopy (TEM) and scanning electron microscopy (SEM). The TEM images demonstrated that OE-MSC-Exos possessed classic cup-shaped structure and SEM images demonstrated that the exosomes were spherical in shape (Fig. 1a and b). The particle sizes of OE-MSC-Exos were further determined by nanoparticle tracking analysis (NTA), which ranged from 50 to 150 nm (Fig. 1c). To investigate the chemical components of the exosomes, LC-MS/MS proteomic analysis of OE-MSC-Exos were performed and several classic markers of exosomes such as CD81, CD9, CD63, CD44, CD90 and HSP72 were found in OE-MSC-Exos (Fig. S1). To further identify the source of exosomes and provide a basis for future applications [34], common surface markers of exosomes, such as the tetraspanin protein family (CD63 and CD9) and mesenchymal stem cell surface markers (CD44, CD90 and CD27), were analyzed by western blotting and flow cytometry. As shown in Fig. 1d, CD63, CD9, TSG101 and ALIX were positive, while calnexin, a cellular marker, was negative in OE-MSC-Exos, confirming that exosomes were derived from mesenchymal stem cells. Then, the immunophenotype of OE-MSC-Exos was determined. Homologous labeling of OE-MSCs, such as CD44, CD90, and CD29, was expressed on these exosomes, whereas hematopoietic cell markers, such as CD34 and CD45, and the myeloid cell marker CD11b were absent (Fig. 1e). Furthermore, more than 85% of the exosomes expressed CD63, indicating that the membrane antigens of OE-MSC-Exos are preserved with high purity (Fig. S2).

It has been recently reported that Tfh cells can facilitate the differentiation of germinal center (GC) B cells into plasma cells, which leads to RA pathogenesis [7, 9, 35]. Thus, the capacity of OE-MSC-Exos on regulating the Tfh cell polarization was investigated by culturing Naïve T cells with 30, 60, or 90 µg/mL OE-MSC-Exos under Tfh polarization conditions respectively. As shown in Fig. 1f, OE-MSC-Exos effectively decreased the frequencies of Tfh cells in vitro, indicating that OE-MSC-Exos can successfully inhibit Tfh polarization of Naïve T cells and may restrain pathogenic Tfh cell generation in RA.

### PD-L1 expression of OE-MSC-Exos on suppressing tfh cells via PI3K/AKT pathway

**Fig. 2:**
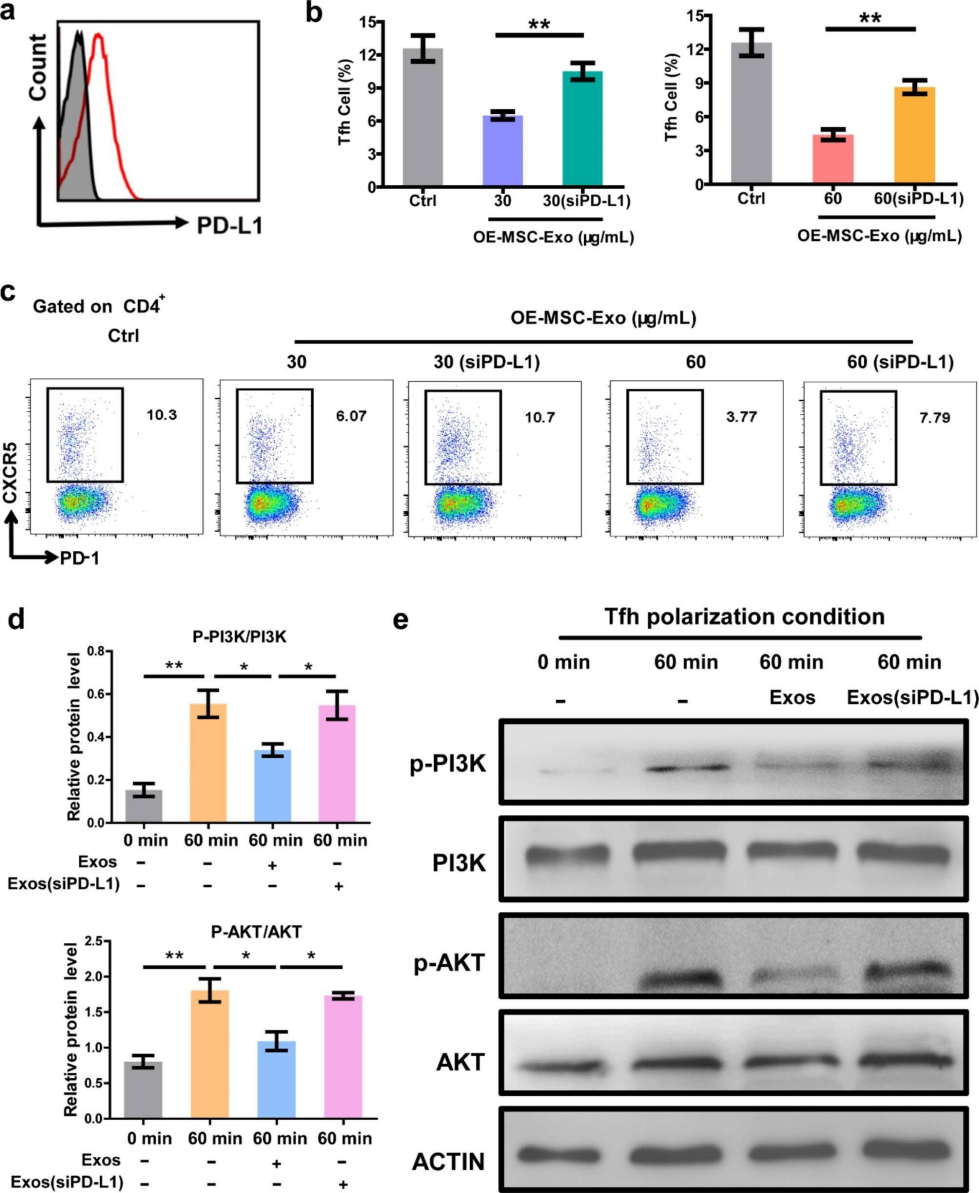
PD-L1 expression on OE-MSC-Exos and mechanisms of OE-MSC-Exos on suppressing Tfh cell polarization. (a) Flow cytometry analysis of PD-L1 expression on the surface of OE-MSC-Exos. (b, c) Tfh cell polarization in the presence of OE-MSC-Exos or OE-MSC-Exos (siPD-L1) after 72 h analyzed by flow cytometry assay. Values represent means ± S.D. (n = 3). (d, e) The phosphorylation levels of PI3K and AKT in naïve T cells treated with OE-MSC-Exos or OE-MSC-Exos (siPD-L1) under Tfh polarization conditions. Values represent means ± S.D. (n = 3). *P < 0.05, **P < 0.01

Numerous studies have reported that the PD-1/PD-L1 pathway can regulate T cell activation, tolerance and exhaustion [36]. Tfh cells highly express the inhibitory PD-1 molecule, and the PD-1/PD-L1 pathway has been shown to to suppresses Tfh cell polarization [37]. Therefore, to understand the mechanism of this immunosuppressive effect, the present of PD-L1 on the surface of OE-MSC-Exos was investigated. As shown in Fig. 2a, flow cytometry analysis showed that PD-L1 was highly expressed on OE-MSC-Exos. Then, whether PD-L1 on MSC-Exos contributes immunosuppressive effect on Tfh polarization was further determined by culturing T cell with OE-MSC-Exos interfered with siPD-L1 (Fig. S4). Importantly, the inhibition of Tfh cell polarization was significantly reversed in the OE-MSC-Exos group treated with siPD-L1, indicating that PD-L1 expressed on OE-MSC-Exos plays a key role in the suppression of Tfh cell differentiation (Fig. 2b and c).

It has been reported that the differentiation of Tfh cells is initiated by activating the PI3K/AKT pathway, which further induces CXCR5 expression [38]. Therefore, to explore the underlying mechanism of immunosuppressive effect on Tfh cells by the released exosomes, T cells were incubated with OE-MSC-Exos or OE-MSC-Exos with siPD-L1 interference, and the protein level of phosphorylated PI3K/AKT in T cells was assessed. As shown in Fig. 2d and e, the protein levels of both phosphorylated PI3K and phosphorylated AKT were significantly decreased in the OE-MSC-Exos treated group, whereas this effect was almost abolished after knocking down PD-L1 expression. These results show that the released exosomes express PD-L1 to inhibit Tfh polarization by down-regulating the PI3K/AKT pathway.

### Fabrication and characterization of OE-MSC-Exos loaded hydrogel

**Fig. 3:**
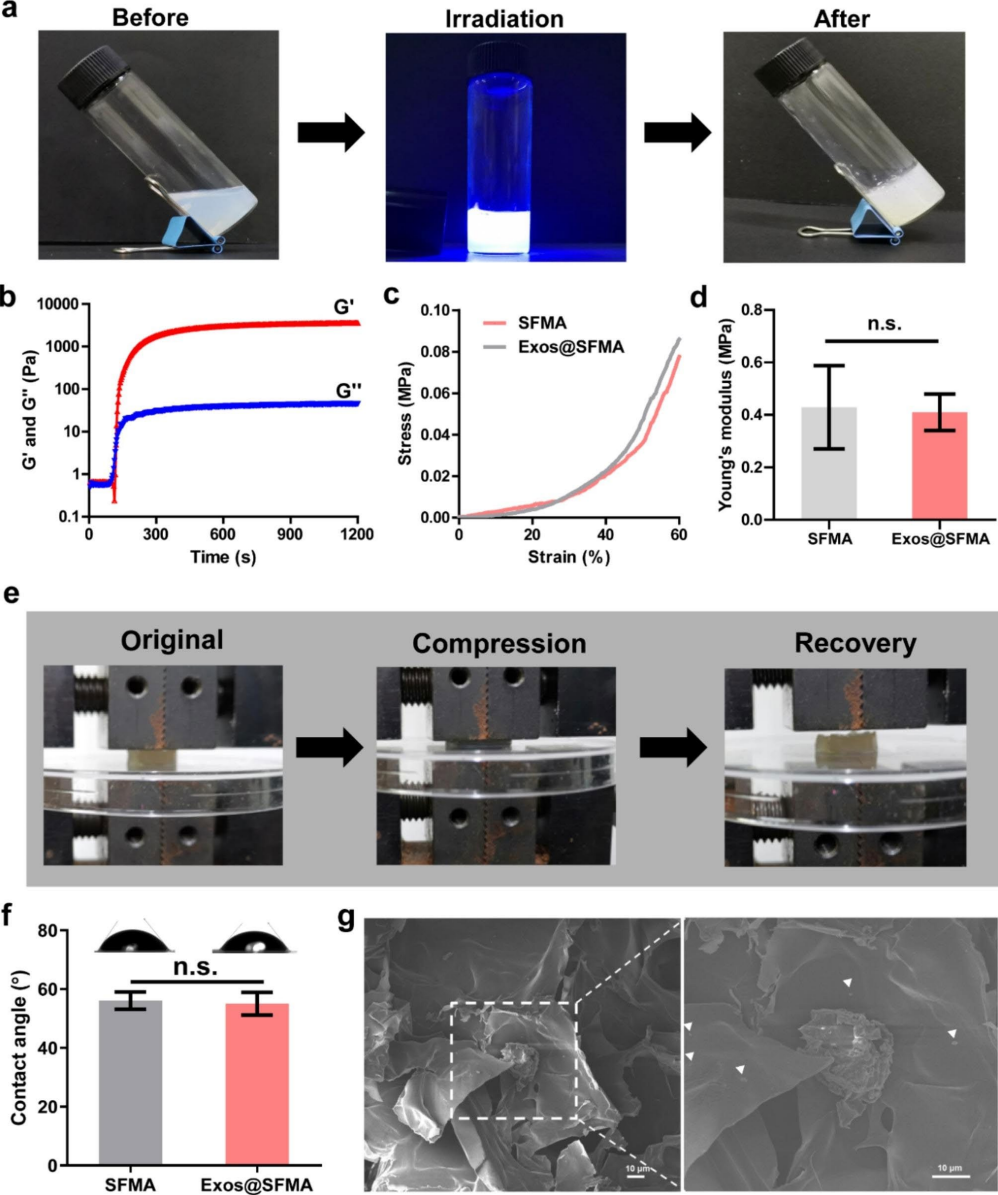
Fabrication and characterization of OE-MSC-Exos loaded hydrogel (Exos@SFMA). (a) Photographs of the photo-triggered gelation process of hydrogel for OE-MSC-Exos encapsulation. (b) Rheological analysis of hydrogels. (c) Compressive stress and (d) Young’s modulus of hydrogels. Values represent means ± S.D. (n = 3). (e) Photographs of compression and recovery process of hydrogels for stress dispersion. (f) Contact angle of hydrogels. Values represent the means ± S.D. (n = 5). (g) SEM images of microstructure and OE-MSC-Exos encapsulated in hydrogels. (Dispersed exosomes are indicated by white arrows) *P < 0.05, **P < 0.01, ***P < 0.001

As encapsulating exosomes within the hydrogel network can effectively prolong exosome release and consumption for enhancing therapeutic efficacy [39, 40], OE-MSC-Exos were loaded into silk fibroin based photo-crosslinkable hydrogel (SFMA). Photographs in Fig. 3a show that the successful photo-triggered gelation of the precursor solution containing 100 µg/mL of OE-MSC-Exos after irradiation to give Exos@SFMA hydrogel. The rapid gelation was achieved within 200 s upon irradiation of 365 nm light (Fig. 3b and S3), which is essential for precious exosome delivery to target sites. As joint destruction usually occurs during RA progression, ideal injectable biomaterials should also possess flexible mechanical properties to distribute the stress of normal physiological activities to protect joints from further damage [41]. As shown in Fig. 3e, a cylindrical sample of Exos@SFMA hydrogel exhibited promising capacity on recovering to its initial shape rapidly from compression when the loading was released, due to the high mechanical strength of silk fibroin, which facilitates the stress dispersion in lesions of RA. Meanwhile, there were no statistically significant differences in the compressive stress and Young’s modulus of hydrogels between SFMA and Exos@SFMA hydrogels, eliminating the effect of exosome encapsulation on the mechanical properties of the silk fiborin based hydrogel system (Fig. 3c and d). Additionally, both hydrogels showed good surface hydrophilicity, which is beneficial for lubrication of the joint (Fig. 3f) [42, 43]. Hydrogen bonds can be formed between phospholipids of exosomes and protein chains within the protein-based hydrogels to facilitate exosome encapsulation [44]. To further demonstrate the successful exosome encapsulation in hydrogels, lyophilized Exos@SFMA hydrogels were observed using SEM. Monodisperse or aggregated exosomes existed on the surface of the porous structure inside hydrogels (Fig. 3g).

### In vitro cell cytotoxicity of the hydrogels

**Fig. 4:**
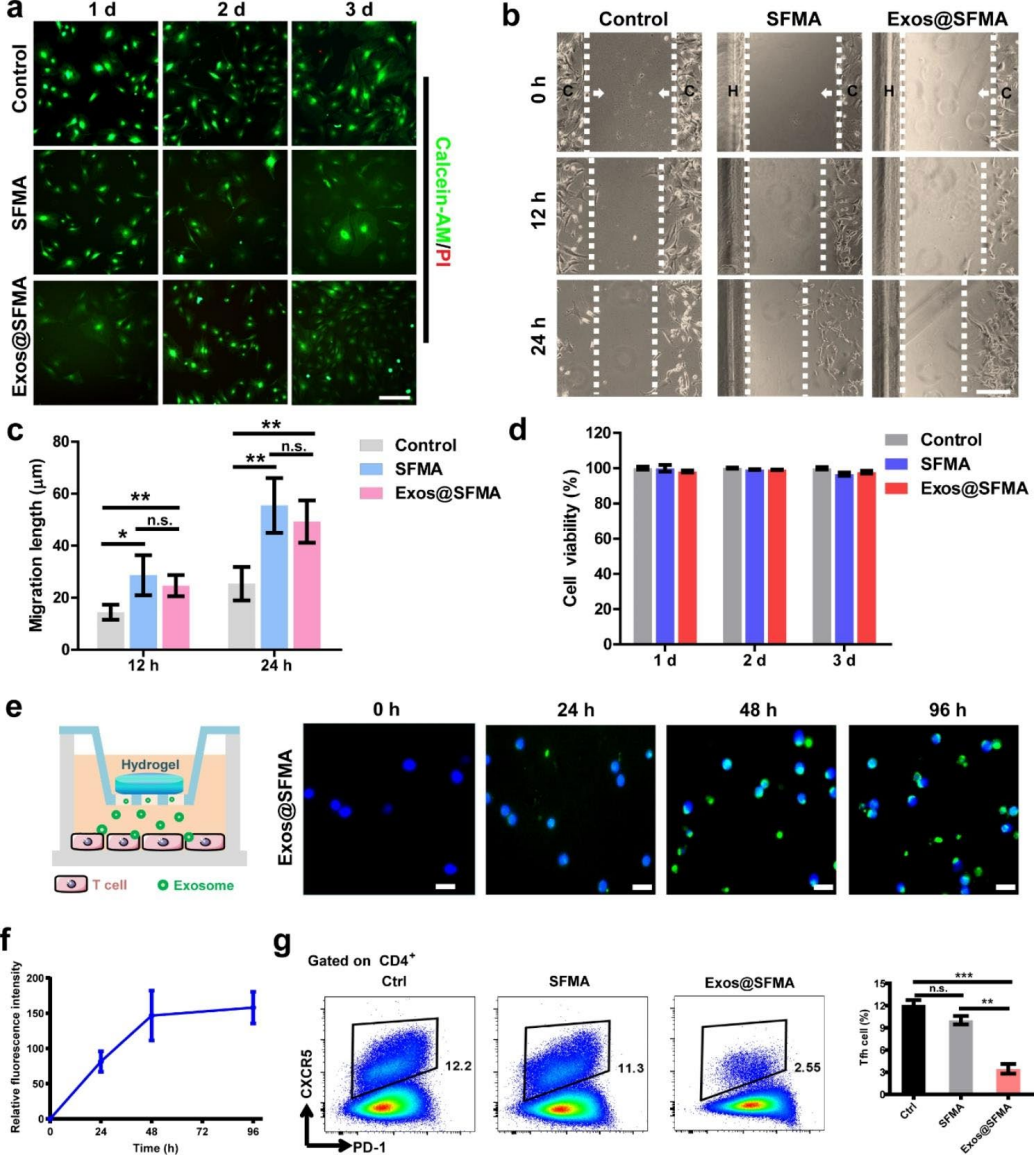
In vitro cell cytotoxicity and recruitment effect of Exos@SFMA on bone marrow stromal cells (BMSCs) and in vitro immunosuppressive effect of Exos@SFMA on Tfh cell polarization. (a) Live/dead assay of BMSCs cultured on hydrogels for 3 days. Scale bar: 100 μm. (b, c) Migration of BMSCs toward Exos@SFMA. (C: Area where BMSCs were seeded; H: Area where hydrogels were fabricated; Arrow: Direction of BMSCs migration) scale bar: 200 μm. Values represent means ± S.D. (n = 3). (d) Cell viability of L929 cells cultured on hydrogels. Values represent means ± S.D. (n = 3). (e) Schematic illustration of the transwell assay and the accumulation of PKH67-labeled OE-MSC-Exos (green) released by Exos@SFMA hydrogel in T cells after 24, 48, 72 h of incubation. (bar = 10 μm). (f) Relative green fluorescence intensity of T cells after 24, 48, 96 h of incubation. Values represent means ± S.D. (n = 4). (g) The proportions of Tfh cells cultured on Exos@SFMA hydrogel after 72 h. Values represent means ± S.D. (n = 3). *P < 0.05, **P < 0.01, ***P < 0.001

To evaluate the in vitro cell cytotoxicity of the hydrogels, L929 fibroblasts were seeded on SFMA and Exos@SFMA hydrogels and assessed by CCK-8 assay. Figure 4d shows that L929 fibroblasts exhibited normal proliferation with cell viability of more than 95% during 3 days of incubation on both SFMA and Exos@SFMA hydrogels. Bone marrow stromal cells (BMSCs) are important for cartilage regeneration [20, 45, 46]. The results of Live/Dead staining assay showed that very few dead BMSCs were observed after culturing on the Exos@SFMA hydrogel (Fig. 4a), suggesting that Exos@SFMA hydrogel has excellent cell compatibility to BMSCs for facilitating cartilage repair. Moreover, as shown in Fig. 4b and c, the migration of BMSCs was accelerated by hydrogels and the cells in SFMA and Exos@SFMA group exhibited higher migration rates than control group within 24 h, demonstrating Exos@SFMA holds strong capacity on recruiting BMSCs .

As hydrogels can maintain the bioactivity of exosomes and allow extended exosome release [47], the cellular uptake of the exosomes from Exos@SFMA hydrogel by T cells was investigated. The OE-MSC-Exos were labelled with membrane dye PKH67 and encapsulated in the hydrogel. As shown in Fig. 4e and f, green fluorescence labelled exosomes were constantly released from Exos@SFMA and gradually accumulated in T cells within 96 h of incubation, demonstrating the prolonged exosome release and consumption. Then, immunosuppressive effect of hydrogels on Tfh polarization was evaluated by transwell assays. Significantly, Exos@SFMA hydrogels effectively suppressed T cell activation by releasing exosomes after 48 h of incubation, whereas no immunosuppressive effect on Tfh cells was found for the pure SFMA hydrogel (Fig. 4g). These results indicate that the Exos@SFMA hydrogel can provide the sustained release of exosomes and exhibits an excellent capacity on suppressing Tfh polarization.

### Exos@SFMA efficiently alleviated the progression of CIA

**Fig. 5:**
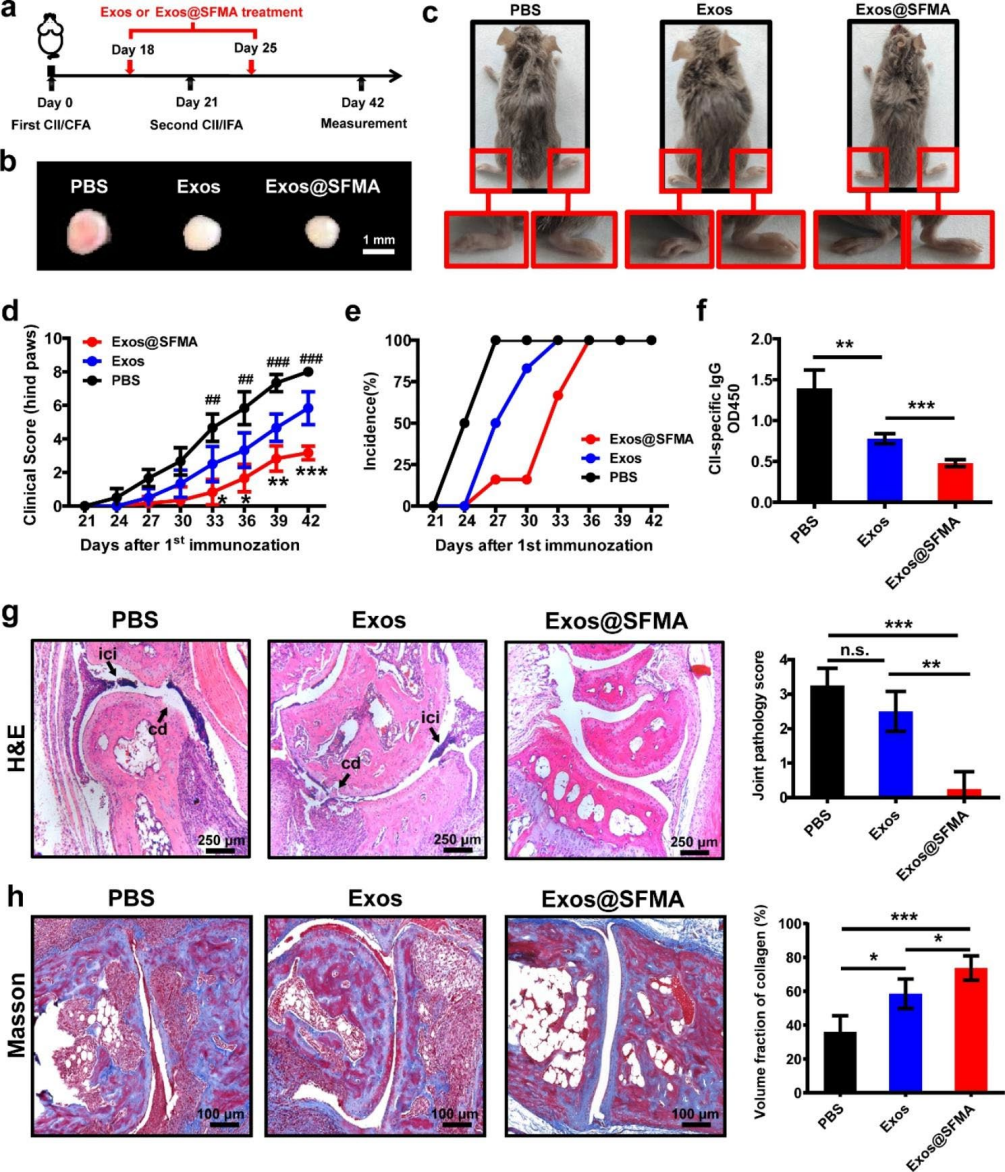
Exos@SFMA efficiently alleviated the progression of CIA and modulated immune dysregulations. (a) Schematic illustration of establishing the CIA mouse model and OE-MSC-Exo or Exos@SFMA treatments. (b, c) Photographs of popliteal lymph nodes (b) and the hind paws (c) of mice treated with PBS, OE-MSC-Exos or Exos@SFMA on Day 42 after the first immunization. (d, e) Clinical score (d) and incidence (e) in CIA mice from each group monitored every 3 days after the first immunization. Values represent means ± S.D. (n = 6). (f) Serum levels of CII-specific autoantibodies from each group measured using ELISA. Values represent means ± S.D. (n = 4). (g) Hematoxylin and eosin staining of hind paw sections from each group on day 42 after the first immunization. Cartilage destruction (cd) and inflammatory cell infiltration (ici) are indicated by black arrows. Values represent means ± S.D. (n = 4). (h) Expression of collagen (blue) Masson’s trichrome staining assay. Values represent means ± S.D. (n = 5). *P < 0.05, **P < 0.01, ***P < 0.001

Inspired by the positive results of in vitro studies, the therapeutic effect of the Exos@SFMA hydrogel on treating RA was evaluated. The collagen-induced arthritis (CIA) mouse model was established by immunization with CII/CFA on Day 0 and boosted with CII/IFA on Day 21. The hind paws were treated with OE-MSC-Exos (Exos group) or in situ formed Exos@SFMA hydrogel (Exos@SFMA group) on Day 18 and 25 after the first immunization (Fig. 5a). As shown in Fig. 5b and c, the degree of swelling in each paw was significantly ameliorated and the size of the draining lymph nodes was also significantly reduced in Exos@SFMA hydrogel treated mice on Day 42 after the first immunization. Moreover, the clinical score was significantly lower than that of other groups and the development of arthritis was strikingly delayed in the Exos@SFMA hydrogel group (Fig. 5d and e). The notable immunological feature in CIA mice is the excessive production of autoantibodies against CII [48]. As shown in Fig. 5f, the level of anti-CII autoantibodies in the serum of mice in Exos@SFMA hydrogel group was significantly lower than those in control group and Exos group. Furthermore, the section of hind paws on Day 42 was evaluated with H&E staining. The histological examination indicated that cartilage destruction (cd) and inflammatory cell infiltration (ici) occurred in the joints of mice in both the PBS and OE-MSC-Exos groups, whereas significant improvement was achieved in the Exos@SFMA group with clear joint spaces, intact articular cartilage and much less joint inflammation (Fig. 5g). Masson’s trichrome staining assay also indicated an increased volume fraction of collagen at articular cartilage in Exos@SFMA group than PBS and Exos groups (Fig. 5h). Taken together, these results suggest that Exos@SFMA holds promising therapeutic effect on the treatment of RA and effectively protects articular cartilage, which is much more efficient than OE-MSC-Exos alone.

### Modulation of immune pathogenesis during RA development

**Fig. 6:**
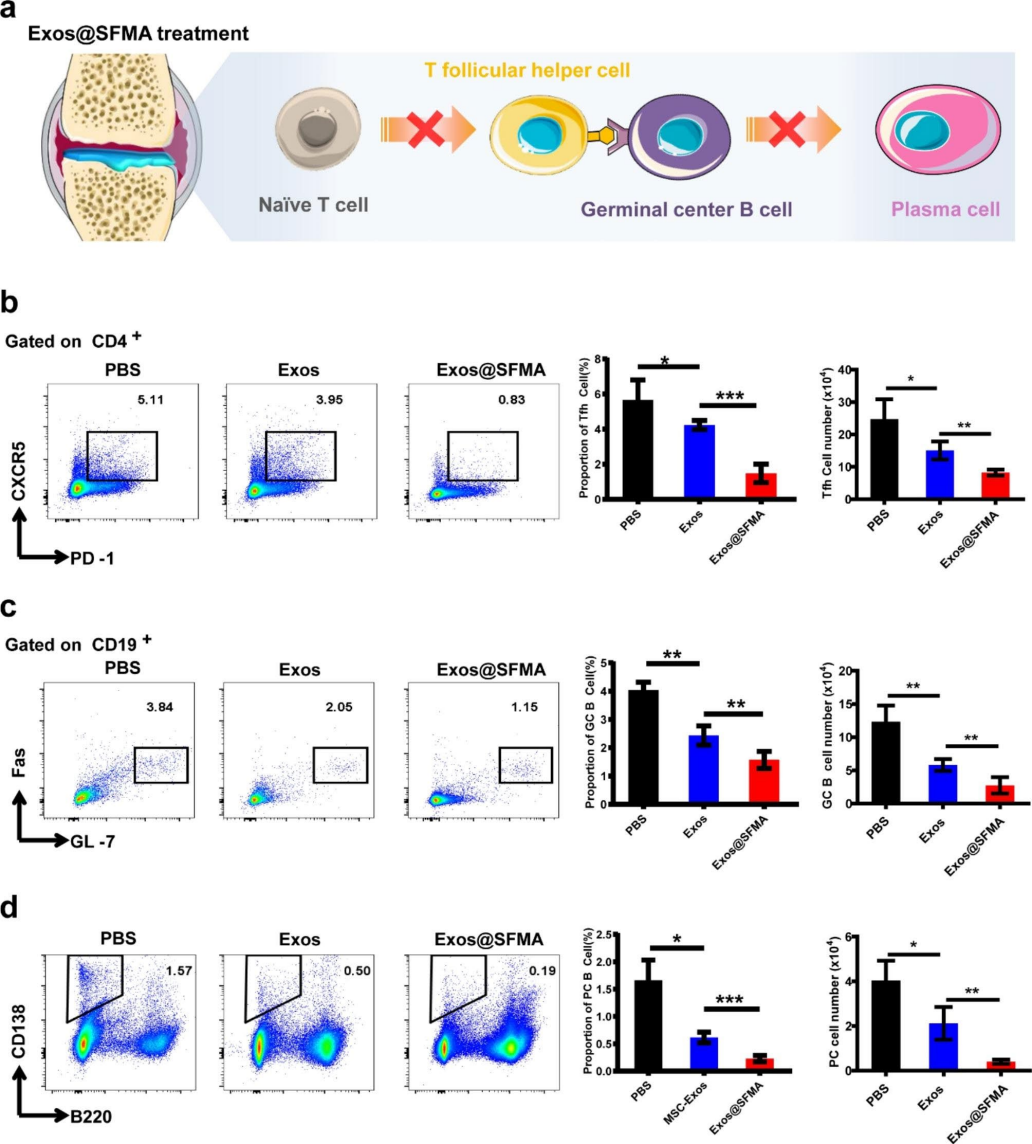
Exos@SFMA efficiently modulated immune dysregulations. (a) Schematic illustration of modulating immune pathogenesis by inhibiting Tfh cell polarization and B cell development in RA via Exos@SFMA treatment. (b-d) Modulation of immune pathogenesis in RA development. Proportions and cell number of Tfh cells (b), germinal center B cells (c), and plasma cells (d) in popliteal lymph nodes (PLN) of CIA mice treated with PBS, OE-MSC-Exos or Exos@SFMA on day 42 after the first immunization. Values represent means ± S.D. (n = 4). *P < 0.05, **P < 0.01

Importantly, as Tfh cells play an essential pathogenic role in RA, the efficacy of Exos@SFMA hydrogel on suppressing the Tfh cell response in RA pathogenesis was explored in CIA mice (Fig. 6a). As shown in Fig. 6b, the percentage of Tfh cells in the popliteal lymph nodes (PLN) of CIA mice was remarkably reduced after treatment with Exos@SFMA hydrogel, which was significantly lower than that in OE-MSC-Exos group. In RA pathogenesis, Tfh cells can further promote the proliferation of GC B cells and their differentiation into plasma cells to damage the joint [7, 9]. Thus, the development of GC B cells and plasma cells in CIA mice after treatment was further investigated. As shown in Fig. 6c and d, the frequencies of both GC B cells and plasma cells in MLNs were also significantly decreased in Exos@SFMA hydrogel group. These results demonstrated that Exos@SFMA hydrogel treatment can effectively modulate the immune pathogenesis of RA, leading to the alleviation of the disease.

## Conclusion

In this work, we demonstrated that OE-MSC-Exos improved hydrogel system held promising immunotherapeutic effects, excellent mechanical property and bioactivities for the effective treatment of RA, which directly targeted immune pathogenesis of RA with enhanced protective effects on inflamed joints. Although MSCs have been demonstrated to display therapeutic effect in RA, the majority of infused stem cells get entrapped in filter organs without significantly homing to sites of injury [49, 50]. Exosomes are essential paracrine products of MSCs, which have emerged as important mediators of cellular and interorgan communication for the replacement of cell-based therapy [15]. Although exosomes are smaller than cells, it contains quite complex biomolecules, including proteins and RNA, which holds enhanced capacity on cell penetrating for delivery of therapeutic biomolecules [51–53]. At present, studies have found that exosomes derived from various MSCs display good therapeutic effect on CIA through microRNA or cytokines (Table S1) [54–59]. In this study, we demonstrated that PD-L1 on OE-MSC-Exos played a critical role in exosome mediated immunosuppression of Tfh cell differentiation, depending on PI3K/AKT pathway. In summary, our work contributes to illustrating the therapeutic mechanism of transplanted stem cell-derived exosomes and provides a powerful platform to treat RA and other autoimmune disorders in the future.

## Experimental section/methods

### Isolation and culture of OE-MSCs and BM-MSCs

For the culture of OE-MSCs, the olfactory epithelium tissue was obtained from the nasal cavity of DBA/1 mice (4-week-old) and cultured in the medium (DMEM/F-12 supplemented with 15% fetal bovine serum) (Gibco) for 7 days. The growth of adherent cells was observed after removal of non-adherent cells. When the adherent cells reached 90% confluence in the flask, they were trypsinized and expanded for three passages.

For the culture of bone marrow mesenchymal stem cells (BMSCs), BMSCs were harvested from the femurs and tibiae of wild-type mice and culturing them in medium (DMEM supplemented with 15% fetal calf serum) (Gibco) for 3 days. Then, nonadherent cells were removed by careful three-time washings with PBS. The adherent cells were expanded for three passages and used for experiments.

### Isolation of OE-MSC-Exos

Isolation of exosomes from OE-MSCs was described previously [13]. Briefly, OE-MSCs were washed three times with PBS and cultured in the medium (DMEM/F-12 supplemented with exosome-depleted fetal bovine serum) for 48 h. The culture supernatants were collected and centrifuged at 300 g for 10 min, 2000 g for 10 min, and 10,000 g for 30 min at 4 °C to remove cells and debris. This was followed by ultracentrifugation spins at 10,000 g (Beckman Coulter, California, USA) for 1 h at 4 °C. The exosomal pellets were washed with PBS and spun 10,000 g centrifugation for another 1 h at 4 °C. Finally, the OE-MSC-Exos were resuspended in PBS and stored at − 80 °C. The protein concentration of OE-MSC-Exos was measured with bovine calf albumin (BCA) kit (CWBIO, Beijing, China). The size of the OE-MSC-Exos was measured by ZetaView PMX 110 (Particle Metrix) and data was analysed using the NTA software ZetaView 8.04.02.

### Uptake of OE-MSC-Exos by T cells

To evaluate the cellular uptake of the released exosomes from Exos@SFMA hydrogel by T cells, OE-MSC-Exos were labeled with the PKH67 Fluorescent Cell Linker Kit (Sigma-Aldrich) and encapsulated the hydrogel in a transwell chamber. Then, it was incubated with T cells by transwell assay for 24, 48 and 96 h, and photographed under an Olympus FluoView FV1000 confocal microscope.

### Immunosuppression of tfh differentiation

Naïve CD4^+^ T cells were purified from the spleens of wild-type mice using naïve CD4^+^ T cell Isolation Kit (Stem Cell). Purified murine naive CD4^+^ T cells (1.75 × 10^6^ /mL) were seeded in a culture plate precoated with anti-CD3 (2 µg/mL) and anti-CD28 (2 µg/mL) antibodies and incubated with OE-MSC-Exos or Exos@SFMA hydrogel under Tfh polarization conditions for 3 days. Cytokines and neutralizing antibodies for Tfh polarization are as follows: recombinant murine IL-6 (25 ng/mL) and IL-21 (20 ng/mL); anti-IFN-γ (5 µg/mL), anti-IL-4 (5 µg/mL) and anti-TGF-β (5 µg/mL) neutralizing antibodies.

In order to explore the effects of PD-L1 molecule carried by OE-MSC-Exos on Tfh differentiation, the proportion of Tfh cells was detected after adding siPD-L1-OE-MSC-Exos into the Tfh induction system. To obtain siPD-L1-OE-MSC-Exos, PD-L1 siRNA (GCCACAGCGAATGATGTTT) and nonspecific scramble siRNA (RiboBio Co, Guangzhou, China) was designed and synthesized. OE-MSCs were transfected with PD-L1 siRNA or negative control using lipofectamine 2000 (Invitrogen) according to the manufacturers’ instructions and exosomes were extracted from transfected cells following the above protocol.

### Animal experiments

DBA/1J mice (8–10 weeks old) were obtained from the Shanghai Laboratory Animal Center (Shanghai, China) and maintained in the Jiangsu University Animal Center (Jiangsu, China). All animal experiments were approved by the Jiangsu University Animal Ethics and Experimentation Committee.

### Arthritis induction and treatment

CIA mice were immunized twice using bovine type II collagen (Chondrex, WA, USA). Bovine type II collagen and Freund’s complete adjuvant (SigmaAldrich, St. Louis, MO) were mixed and injected subcutaneously at the base of the tail in the first immunization. In order to boost immunization, the mixture of bovine type II collagen and Freund’s incomplete adjuvant (SigmaAldrich, St. Louis, MO) were administered 21 days later. To explore the effects of the Exos@SFMA hydrogels treatment, The hind paws were treated with OE-MSC-Exos or in situ formed Exos@SFMA hydrogel on days 18 and 25 after the first immunization. The joint tissue of mice was collected for histologic analyses with H&E staining and Masson’s trichrome staining.

### Statistical analysis

All data were shown as the means ± Standard Deviation (SD). The statistical significance was determined by the Student’s t test or one-way ANOVA. All analyses were performed using SPSS 16.0 software. P values < 0.05 were considered statistically significant.

## Electronic supplementary material

Below is the link to the electronic supplementary material.

Supplementary Material 1
