# Supplementary material for: Exosome inspired photo-triggered gelation hydrogel composite on modulating immune pathogenesis for treating rheumatoid arthritis
Source: J Nanobiotechnology. 2023 Mar 28;21:111. doi: 10.1186/s12951-023-01865-8 (PMC10044428; doi:10.1186/s12951-023-01865-8)
Supplement: Supplementary file 1 — Supplementary Material 1 [file 12951_2023_1865_MOESM1_ESM.docx]

Supporting Information

Exosome inspired photo-triggered gelation system on modulating immune pathogenesis for treating rheumatoid arthritis

Ke Rui^1#^, Xiaoxuan Tang^2#^, Ziwei Shen^1#^, Chao Jiang^1^, Qiugang Zhu^3^, Shiyi Liu^3^, Nan Che^4^, Jie Tian^1,3*,^ Jue Ling^2*^ and Yumin Yang^2*^

^1^Institute of Medical Immunology, Affiliated Hospital of Jiangsu University, Zhenjiang, China

^2^Key Laboratory of Neuroregeneration, Ministry of Education and Jiangsu Province, Co-innovation Center of Neuroregeneration, Jiangsu Clinical Medicine Center of Tissue Engineering and Nerve Injury Repair, Nantong University, Nantong, China

^3^Department of Immunology, Jiangsu Key Laboratory of Laboratory Medicine, School of Medicine, Jiangsu University, Zhenjiang, China

^4^Department of Rheumatology, The First Affiliated Hospital of Nanjing Medical University, Jiangsu, China

*Corresponding Author

Jie Tian, tianjie@ujs.edu.cn

Jue Ling, jl2016@ntu.edu.cn

Yumin Yang, yangym@ntu.edu.cn

#These authors contributed equally.

**1. Materials and methods**

***1.1. Flow cytometric analysis***

For evaluation of surface markers, Single cell suspensions were stained with fluorescence-labeled monoclonal antibodies (mAbs) specific for the following phenotypic markers: anti-CD4 (RM4-5), anti-PD-1 (29F.1A12), anti-PD-L1 (MIH5), anti-B220 (clone RA3-6B2), anti-CD138 (clone 281-2), anti-CD19 (clone 6D5), anti-GL-7 (clone GL7) anti-Fas (clone SA367H8), anti-CXCR5 (SPRCL5) from eBioscience. The stained cells were analyzed with a BD FACSCanto II (Becton Dickinson, NJ, USA) and FlowJo software (TreeStar, Ashland, OR).

To detect OE-MSC-Exos surface markers, latex microspheres (4µm, Thermo) were mixed with OE-MSC-Exos and incubated for 15 min. After washing with PBS, glycine (1M) was added and incubated for 30 min. After washing with PBS (supplemented with 0.5% bull serum albumin), then stained with relevant fluorochrome-conjugated monoclonal antibodies (mAbs): anti-CD29 (TS2/16), anti-CD90 (5E10), anti-CD44 (IM7), anti-CD34 (RAM34) and anti-CD45(30-F11) from eBioscience; anti-CD11b (clone M1/70) from Biolegend. The results were analyzed by flow cytometry.

***1.2. Fabrication of exosome-loaded hydrogels (Exos@SFMA)***

SFMA macromer were synthesized according previous references [1]. Briefly, 10.0 g of mulberry silk was in 50 mL of 9.3 M LiBr (Meryer, China) solution with 0.5% Na_2_CO_3_. Then, 2.0 mL of methacrylic anhydride was added to the mixture and reacted at 60°C for 3 h. The mixture was dialyzed against distilled water at 4°C for 3 days and lyophilized.

To fabricate the exosome-loaded hydrogel, SFMA macromer (20% (w/v)) were dissolved in PBS containing 100 µg/mL of OE-MSC-Exos with 0.5% (w/v) 2-hydroxy-1-(4-(hydroxyethoxy)phenyl)-2-methyl-1-propanone (Irgacure 2959, CIBA Chemicals, Basel, Switzerland) and exposed under 30 mW/cm^2^ UV light (365 nm) for 5 min to obtain Exos@SFMA. The encapsulation of exosomes and inner microstructure were characterized by SEM using an S-3400N II (Hitachi, Tokyo, Japan)

***1.3. Rheological analysis***

Time-sweep oscillatory tests of hydrogels were performed using a Haake MARS rotational rheometer (Thermo Fisher Scientific) with irradiation of 30 mW/cm^2^ UV light (365 nm) at 25°C.

***1.4. Mechanical evaluation***

The stress-strain curve was measured by an electronic universal testing machine (UTM; TFW-58; Shanghai Tuofeng Instrument Technology Co. Ltd., Shanghai, China) under the compression test at a displacement rate of 5 mm/min until the hydrogel sample compressed to 60% and Young's modulus was obtained by calculating the slope of the stress-strain curve.

***1.5. Contact-angle measurement***

Contact angle were measured using a JY-PHA instrument (Chengde Jinhe Instrument Manufacturing Co. Ltd., Chengde, China). Hydrogels were placed on a flat table. Then single-distilled water was added onto the surface of the hydrogel and photographed.

***1.6. Cell cytotoxicity***

Mouse fibroblast (L929) cells (Cell Bank of the Chinese Academy of Sciences, China) were seeded respectively on SFMA hydrogels and Exos@SFMA hydrogels at a density of 5 x 10^4^ cells/ well coating in 24-well plates and incubated for 1, 2 and 3 days at 37 °C. The cytotoxicity of the hydrogels on L929 fibroblast cells was assessed by the cell counting kit-8 (CCK-8) (Beyotime Biotechnology, China). The absorbance of each well was measured at 450 nm by an ElX-800 microelisa reader. Relative cell viability was calculated as following equation: Cell viability (%) = Abs _experiment_/Abs _control_ x 100 %. For the cell viability of BMSCs, the BMSCs were cultured with a density of 5 x 10^4^ cells/well on hydrogels for 3 days in 24-well plates and stained with LIVE/DEAD viability/cytotoxicity kit (Molecular Probes, Eugene, OR, USA), then photographed using a fluorescence microscope at day 1, 2, and 3.

***1.7. BMSCs mirgration***

BMSCs (5 x 10^4^ cells/well) were seeded into each well of Culture-Inserts 2 well (ibidi) coated with/without hydrogels and allowed to incubate at 37 °C for 6 h. Subsequently, the culture medium was replaced with fresh culture medium and the Culture-Inserts were removed to allow migration of BMSCs. Then cell migration towards hydrogels was imaged using microscope (DM2500, Leica, Germany) at 0, 12, 24 hours.

***1.8. Western blot analysis***

Naïve CD4^+^ T cells were negatively purified from wild-type mice and culture under Tfh polarization conditions. Protein extracted from cells was prepared as previously described [1]. The protein from collected cells were separated by 12% SDS-PAGE and transferred to polyvinylidene fluoride (PVDF) membranes (Bio-Rad, CA, USA). The following antibodies were used: anti-PI3K, anti-pPI3K, anti-AKT, and anti-pAKT (Cell Signaling Technology).

***1.9. Clinical score***

From Day 21 of the experiment, mice were scored for clinical signs of arthritis [2]. as follows: 0=no evidence of erythema and swelling; 1=erythema and mild swelling confifined to the tarsals or ankle joint; 2=erythema and mild swelling extending from the ankle to the tarsals; 3=erythema and moderate swelling extending from the ankle to metatarsal joints; 4=Erythema and severe swelling encompass the ankle, foot and digits, or ankylosis of the limb.

***1.10. Histopathological assessment***

Mouse joint specimens were fixed with 10% paraformaldehyde for 3 days. The joint section with thickness of 4 μm were prepared and stained with haematoxylin and eosin (H&E) for morphologic examination. The sections were analysed based on the degree of cell infiltration, cartilage damage, pannus formation and bone erosion to assess the severity of tissue damage. The sections were analysed using the following scoring system: 0= normal; 1=mild (some exudate in joint space or mild synovial changes); 2=moderate(exudative synovitis evident in >1 area, early pannus formation, cartilage generally OK or patchy change only); 3=moderately severe(changes between grade 2 and 4); 4=severe(widespread exudative synovitis with pannus and cartilage changes and some bone erosion); 5=Very severe (joint destruction or ankylosis with thick synovitis) [3].

***1.11. Autoantibody evaluation***

The levels of Serum bovine type II collagen-specific antibody were measured via sandwich enzyme-linked immunosorbent assay (ELISA). Briefly, 96-well plates were pre-coated with bovine type II collagen (5 μg/mL) at 4 °C overnight. The plate was washed and blocked with PBS (supplemented with 1% bull serum albumin) for 1h at room temperature. Coated plates were added by the diluted serum samples and incubated at room temperature for 2 h, followed by incubation of HRP-conjugated goat anti-mouse IgG Abs at room temperature for 2 h. After washing, the TMB substrate was added and incubated for 30 min. Then, stop solution was added and absorbance was measured using a microplate reader (BioTek, Winooski, USA) at 450 nm.

***1.12. LC-MS/MS analysis***

OE-MSC-Exos were lysed in STD buffer and centrifuged at 1000 × g for 10 min to collect the supernatants. Proteins were identified using a Q Exactive Orbitrap LC-MS/MS system (Thermo Fisher Scientific, MA, USA).


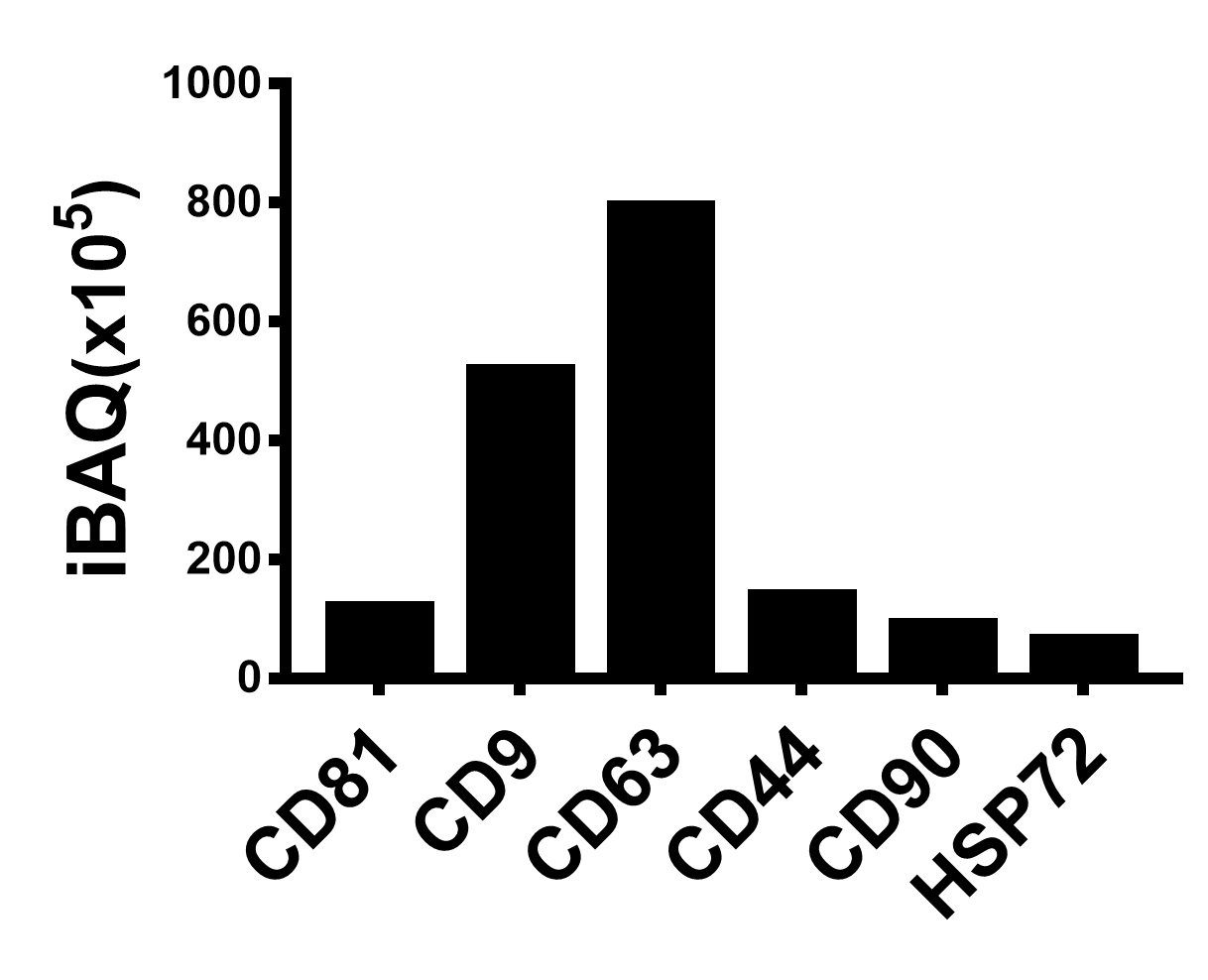


**Fig. S1** LC-MS/MS proteomic analyses of OE-MSC-Exos.

**
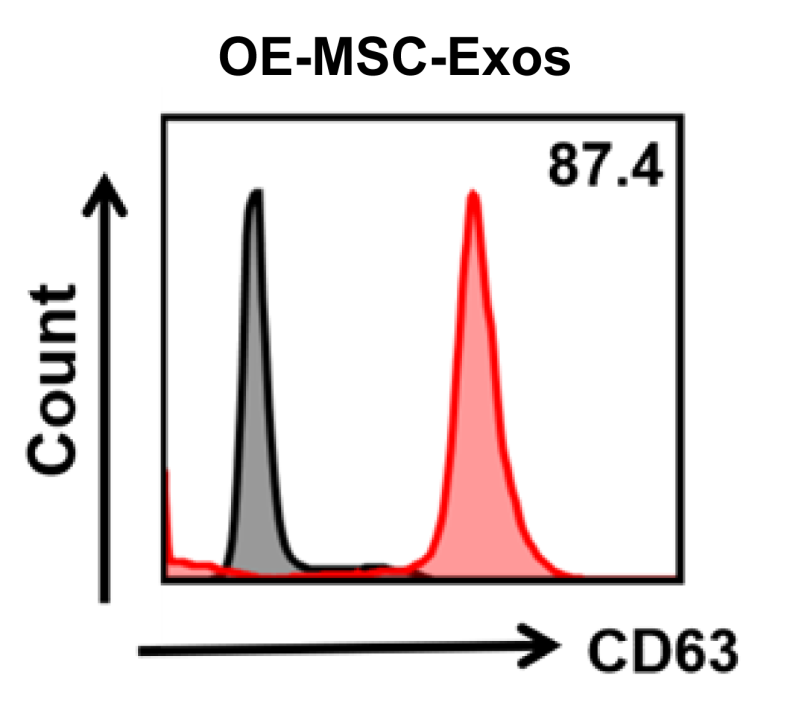
**

**Fig. S2** Flow cytometry analysis of CD63 expression on the surface of OE-MSC-Exos.

**Fig. S3** Gel points of hydrogels. Values represent means ± S.D. (n = 3)

**
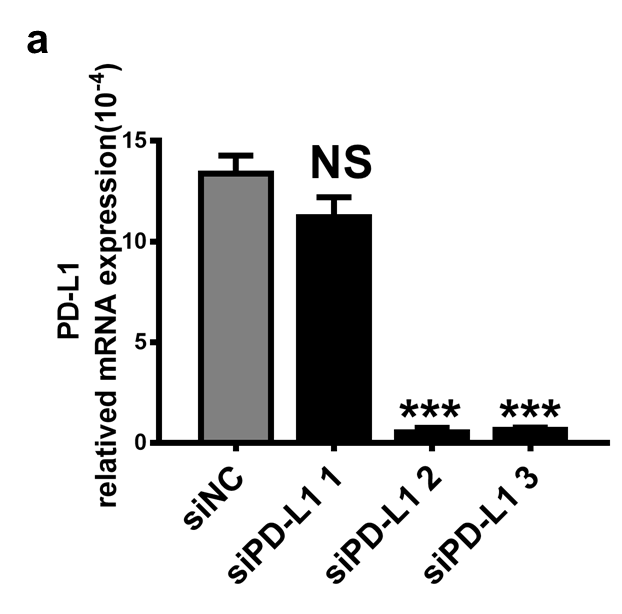
**

**
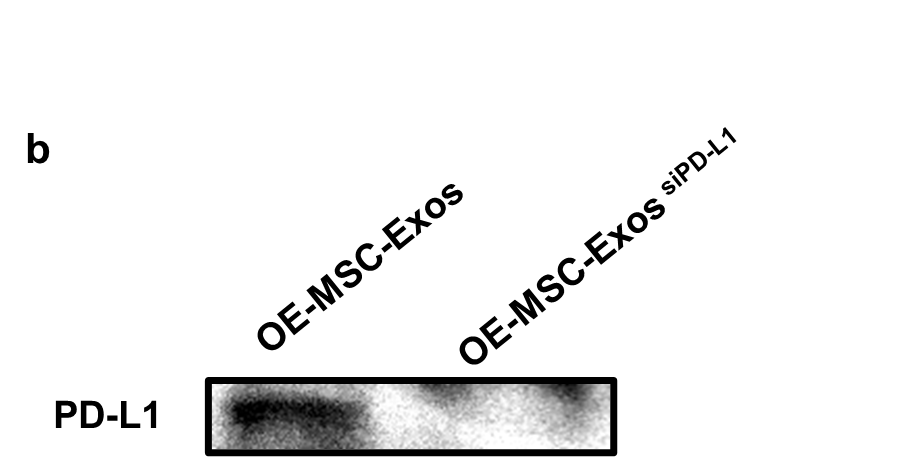
**

**Fig. S4** The interference efficiency of PD-L1 in OE-MSC-Exos. (a) The mRNA levels of PD-L1 in OE-MSCs interfered with siRNA or negative control were analyzed by qRT-PCR. (b) Western blot was used to analyze the level of PD-L1 in OE-MSCs-Exos from PD-L1-silenced OE-MSCs. Values represent means ± S.D. (n = 3). ****p* < 0.001

**Table S1. The role of MSC-Exosomes in the treatment of CIA**

| **Exosomes sources** | **Exosomesreactive molecules** | **Target cells/ tissue** | **Mechanism of action** | **Effect** | **Ref** |
| --- | --- | --- | --- | --- | --- |
| BM-MSCs | miRNA-150-5p | Fibroblast-like synoviocytes (FLS) | Targets MMP14 and VEGF2 | Decreases migration and invasion in FLS and downregulates tube formation in HUVECs | [4] |
| BM-MSCs | miRNA-320a | FLS | Suppresses CXCL9 expression | Attenuates arthritis and bone damage | [5] |
| BM-MSCs | miRNA-192-5p | FLS | Inhibits the levels of pro-inflammatory factors and suppresses synovial hyperplasia by RAC2 | Delays the event of the inflammatory response | [6] |
| BM-MSCs | unspecified | B cell | Expands Breg cells and decreases plasmablast differentiation | Lowers disease incidence and deceases clinical score  Reduces levels of serum auto-antibodies | [7, 8] |
| OE-MSCs | PD-L1 | Tfh cells | Suppresses PD-1 expression | Effectively relieved synovial inflammation and joint destruction | This work |
| Gingival- MSCs | IL- 10 | Th17 cells | Inhibiting IL-17RA-NF-κB signal pathway | Alleviate the inflammation and bone erosion | [9] |

**References**

1. Tang X, Gu X, Huang T, Chen X, Zhou Z, Yang Y, Ling J (2021) Anisotropic silk-inspired nerve conduit with peptides improved the microenvironment for long-distance peripheral nerve regeneration. ACS Macro Lett 10:1501-1509. https://doi.org/10.1021/acsmacrolett.1c00533

2. Brand DD, Latham KA, Rosloniec EF (2007) Collagen-induced arthritis. Nat Protoc 2:1269-1275. https://doi.org/10.1038/nprot.2007.173

3. Esvaran M, Conway P (2019) *Lactobacillus fermentum* PC1 has the capacity to attenuate joint inflammation in collagen-induced arthritis in DBA/1 mice. Nutrients 11:785. https://doi.org/10.3390/nu11040785

4. Chen Z, Wang H, Xia Y, Yan F, Lu Y (2018) Therapeutic Potential of Mesenchymal Cell-Derived miRNA-150-5p-Expressing Exosomes in Rheumatoid Arthritis Mediated by the Modulation of MMP14 and VEGF. J Immunol 201:2472-2482. https://doi.org/10.4049/jimmunol.1800304

5. Meng Q, Qiu B (2020) Exosomal MicroRNA-320a Derived From Mesenchymal Stem Cells Regulates Rheumatoid Arthritis Fibroblast-Like Synoviocyte Activation by Suppressing CXCL9 Expression. Front Physiol 11:441. https://doi.org/10.3389/fphys.2020.00441

6. Zheng J, Zhu L, Iok In I, Chen Y, Jia N, Zhu W (2020) Bone marrow-derived mesenchymal stem cells-secreted exosomal microRNA-192-5p delays inflammatory response in rheumatoid arthritis. Int Immunopharmacol 78:105985. https://doi.org/10.1016/j.intimp.2019.105985

7. Cosenza S, Toupet K, Maumus M, Luz-Crawford P, Blanc-Brude O, Jorgensen C, Noel D (2018) Mesenchymal stem cells-derived exosomes are more immunosuppressive than microparticles in inflammatory arthritis. Theranostics 8:1399-1410. https://doi.org/10.7150/thno.21072

8. Cosenza S, Ruiz M, Toupet K, Jorgensen C, Noel D (2018) Mesenchymal stem cells derived exosomes and microparticles protect cartilage and bone from degradation in osteoarthritis. Sci Rep 7:16214. https://doi.org/10.1038/s41598-017-15376-8

9. Tian X, Wei W, Cao Y, Ao T, Huang F, Javed R, Wang X, Fan J, Zhang Y, Liu Y, Lai L, Ao Q (2022) Gingival mesenchymal stem cell-derived exosomes are immunosuppressive in preventing collagen-induced arthritis. J Cell Mol Med 26:693-708. https://doi.org/ 10.1111/jcmm.17086
